# Multidimensional 3D-Printed Scaffolds and Regeneration of Intrabony Periodontal Defects: A Systematic Review

**DOI:** 10.3390/jfb15020044

**Published:** 2024-02-15

**Authors:** Sotiria Davidopoulou, Panagiotis Karakostas, Leonidas Batas, Panagiotis Barmpalexis, Andreana Assimopoulou, Christos Angelopoulos, Lazaros Tsalikis

**Affiliations:** 1 Department of Operative Dentistry, Dental School, Aristotle University of Thessaloniki, 54124 Thessaloniki, Greece; 2 Department of Preventive Dentistry, Periodontology and Implant Biology, Dental School, Aristotle University of Thessaloniki, 54124 Thessaloniki, Greece; karako.dent@gmail.com (P.K.); batas@dent.auth.gr (L.B.); tsalikis@dent.auth.gr (L.T.); 3 Department of Pharmaceutical Technology, School of Pharmacy, Aristotle University of Thessaloniki, 54124 Thessaloniki, Greece; pbarmp@pharm.auth.gr; 4 Organic Chemistry Lab, School of Chemical Engineering, Aristotle University of Thessaloniki, 54124 Thessaloniki, Greece; adreana@auth.gr; 5 Department of Oral Diagnosis & Radiology, School of Dentistry, National and Kapodistrian University of Athens, 10679 Athina, Greece; cangelopou@dent.uoa.gr

**Keywords:** 3D printing, scaffolds, periodontal regeneration, defect

## Abstract

Background: The utilization of regenerative techniques in periodontology involves tailoring tissue engineering principles to suit the oral cavity’s unique environment. Advancements in computer-assisted technology, specifically utilizing cone beam computed tomography (CBCT), enabled the fabrication of 3D-printed scaffolds. The current review aims to explore whether 3D-printed scaffolds are effective in promoting osteogenesis in patients with periodontal defects. Methods: A thorough exploration was undertaken across seven electronic databases (PubMed, Scopus, ScienceDirect, Google Scholar, Cochrane, Web of Science, Ovid) to detect pertinent research in accordance with specified eligibility criteria, aligning with the PRISMA guidelines. Two independent reviewers undertook the screening and selection of manuscripts, executed data extraction, and evaluated the bias risk using the Newcastle–Ottawa Scale for non-randomized clinical trials and SYRCLE’s risk of bias tool for animal studies. Results: Initially, 799 articles were identified, refined by removing duplicates. After evaluating 471 articles based on title and abstract, 18 studies remained for full-text assessment. Eventually, merely two manuscripts fulfilled all the eligibility criteria concerning human trials. Both studies were prospective non-randomized clinical trials. Moreover, 11 animal studies were also included. Conclusions: The use of multidimensional, 3D-printed, customized scaffolds appears to stimulate periodontal regeneration. While the reported results are encouraging, additional studies are required to identify the ideal characteristics of the 3D scaffold to be used in the regeneration of periodontal tissue.

## 1. Introduction

Periodontitis, characterized as a persistent inflammatory condition, is linked to the buildup of dental plaque, impacting tissues that support the teeth, including the periodontal ligament and alveolar bone [1,2,3,4]. Its prevalence is notably elevated among individuals aged 40 years and older [5,6,7]. Moreover, it stands as the leading cause of tooth extraction in adults aged 60 and above [5,8,9]. There are several clinical and radiographic signs that can crucially help the diagnosis, such as bleeding on probing (BOP), deep probing depths (PDs), clinical attachment loss (CAL), presence of purulence, alveolar bone resorption, and mobility, ultimately contributing to potential tooth loss [3,10,11,12,13,14]. Regarding the seriousness of the condition, its likely progression, and the anticipated results of treatment, clinical evaluation alone is inadequate to set the diagnosis and should always be combined with radiographic examination [15]. In everyday practice, the most frequently used radiographs are the periapical, bitewing and panoramic X-rays. However, the above techniques can sometimes be misleading as they depict a two-dimensional version of a three-dimensional (3D) structure. Therefore, they have limitations including projection errors and superimposition of anatomical structures, as well as a lack of visibility in the bucco-lingual direction. This can make it more challenging to distinguish between the lingual and buccal cortical plate and detect certain types of periodontal defects, such as intrabony defects; osseous craters; 1-, 2-, 3-, and 4-walled defects; and furcation involvements [16]. To address these constraints and enhance the precision of evaluating the form of 3D structures, advanced imaging techniques become imperative.

Cone beam computed tomography (CBCT) is an efficient and non-invasive tool, which provides highly precise 3D images of hard tissues. It was first introduced to dentists and maxillofacial surgeons in 1998 by Mozzo et al. [17]. Compared to conventional computed tomography (CT) technology, it provides a high resolution of 3D images with a lower radiation exposure effectiveness and reduced expense [18]. Research contrasting the efficacy of 3D volumetric images with 2D images in identifying artificial bone defects reveals that Cone Beam Computed Tomography (CBCT) demonstrates a sensitivity ranging from 80% to 100% for the detection and categorization of bone defects. In comparison, intraoral radiographs exhibit a sensitivity of 63% to 67%. Furthermore, when juxtaposed with periapical and panoramic images, CBCT displays notable advantages such as the absence of distortion and overlapping, presenting dimensions that align with the actual size [19]. Due to these benefits, the utilization of CBCT has experienced a notable surge, particularly in the preoperative evaluation and strategic planning of intricate surgical procedures [20]. Currently, CBCT imaging is mainly used in dentomaxillofacial surgery, implantology, orthodontics and endodontics, but it is not a standard technique for the diagnosis of periodontal disease. However, it should be used for periodontal diagnosis provided that conventional radiographic methods do not offer the information needed for therapy [21]. One application of CBCT in periodontology can be the diagnosis and treatment of intrabony defects, which requires the 3D evaluation of the morphology, as stated previously. These defects may contain one, two, or three walls and their base is apical to the alveolar bone [22]. They are often linked with the advanced stages of periodontitis, and in such instances, the application of periodontal regenerative treatments can significantly enhance the outlook for the affected teeth. Given the current array of regenerative procedures, the successful regeneration of intrabony defects is achievable, contingent upon the morphology of the defect, encompassing factors such as size, shape, and angle [23]. Numerous publications have highlighted the effectiveness of regenerative approaches regarding periodontal intrabony defects, surpassing traditional techniques like open flap debridement surgery (OFD) [24,25].

**Figure 1:**
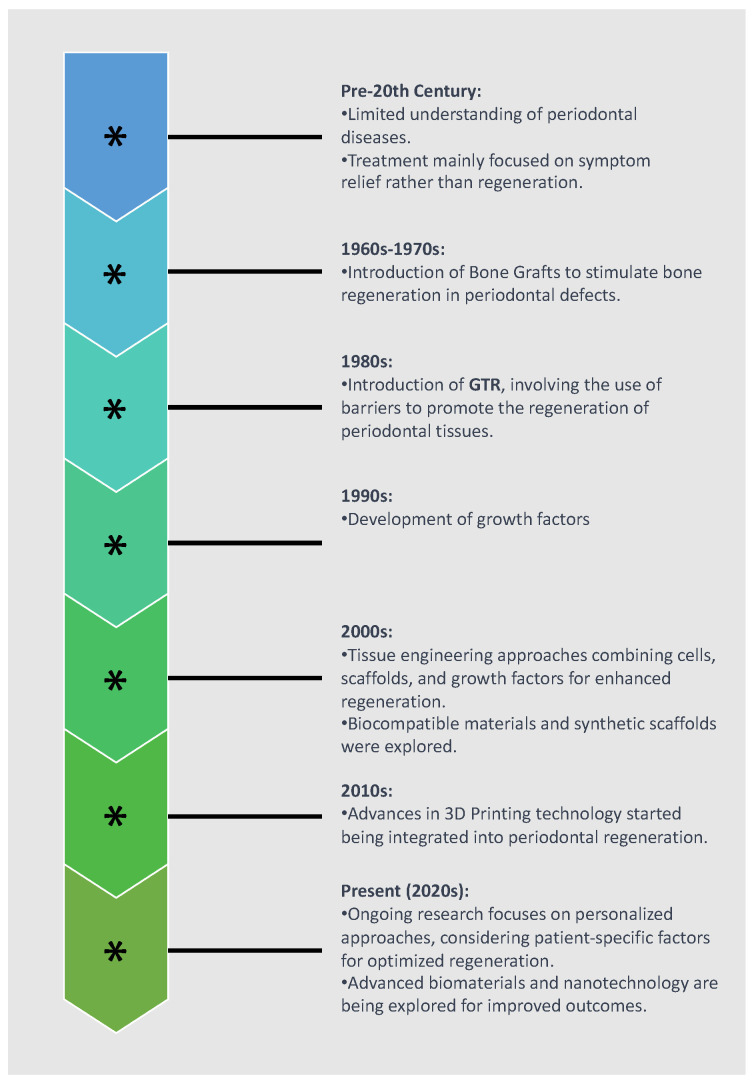
Historical evolution of periodontal regeneration techniques.

**Figure 2:**
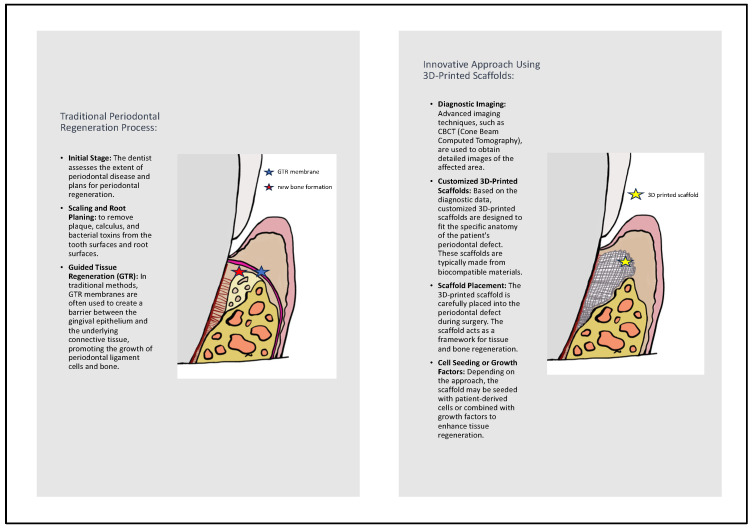
Comparison of traditional periodontal regeneration process and innovative approach using 3D-printed scaffolds.

The utilization of regenerative medicine in periodontology has an extensive historical background, as illustrated in Figure 1. It adheres to the guidelines of personalized tissue engineering customized to suit the distinctive conditions of the oral environment. The ideal scaffold should contain biocompatible oral regenerative cells installed into a porous architecture in order to endure mechanical functions and allow the transportation of essential elements and growth factors which can contribute to healing [26,27]. Figure 2 illustrates the comparative process of traditional periodontal regeneration and the innovative approach incorporating 3D-printed scaffolds. The CAD/CAM technology utilizing the CBCT data has facilitated the manufacture of customized dental products, including polymethyl methacrylate or allogenic bone blocks [28,29,30]. Each block is customized according to the digital signs to precisely match the defect structure, resulting in minimization of the gap between bone and graft which promotes the regenerative result [31,32,33,34]. Given the potential therapeutic benefits that a more comprehensive understanding of these mechanisms could offer, we conducted a systematic review to explore crucial findings related to the efficacy of 3D scaffolds in intrabony defect regeneration across both human and animal subjects. This review is distinctive in its scientific domain as it represents the initial analysis of data derived from studies on humans and animals. 

## 2. Methods

The PRISMA guidelines were followed when conducting the current systematic review [35]. The focus question was: “Are the 3D scaffolds effective in the periodontal intrabony defects regeneration?”.

### 2.1. Eligibility Criteria

The PICOS framework was used as the basis of inclusion and exclusion criteria, as follows:

Population (P): humans with periodontal defects or animals with experimental periodontal defects;
Intervention (I): 3D scaffolds;
Comparison (C): no 3D scaffolds;
Outcome (O): periodontal regeneration, bone augmentation;
Study design (S): randomized controlled clinical trials, case–control observational studies, cohort studies, prospective controlled clinical trials, and animal studies.

Respectively, the exclusion criteria were experimental studies or patients without a history of periodontal disease. Editor’s choices, replies to the author/editor, interviews, commentaries, books/conferences, abstracts, summaries, case reports or reports of cases, narrative reviews, systematic reviews, and meta-analyses were also excluded.

### 2.2. Search Strategy

Two review authors searched independently and systematically in 7 electronic databases (PubMed, Scopus, Science Direct, Google Scholar, Cochrane, Web of Science, Ovid) of articles published up to 30 July 2023. The principal search strategy was:

“periodontal” [All Fields] OR “periodontally” [All Fields] OR “periodontically” [All Fields] OR “periodontics” [MeSH Terms] OR “periodontics” [All Fields] OR “periodontic” [All Fields] OR “periodontitis” [MeSH Terms] OR “periodontitis” [All Fields] OR “periodontitides” [All Fields]) AND (“scaffold” [All Fields] OR “scaffold s” [All Fields] OR “scaffolded” [All Fields] OR “scaffolder” [All Fields] OR “scaffolders” [All Fields] OR “scaffolding” [All Fields] OR “scaffoldings” [All Fields] OR “scaffolds” [All Fields]) AND (“printing, three dimensional” [MeSH Terms] OR (“printing” [All Fields] AND “three dimensional” [All Fields]) OR “three-dimensional printing” [All Fields] OR (“3d” [All Fields] AND “printing” [All Fields]) OR “3d printing” [All Fields].

No limitations were set regarding language, or publication date.

### 2.3. Study Selection

All articles underwent a thorough review process conducted independently by two authors. Human and animal studies meeting the eligibility criteria were assessed at the title, abstract, and full-text level. In cases of discrepancies, these were discussed between two reviewers. If consensus was not obtained, another reviewer was asked to make the final decision. The etiological factors for manuscripts not fulfilling both inclusion and exclusion criteria were documented. The ultimately included studies also underwent data extraction and quality assessment. 

### 2.4. Data Extraction

Primary outcomes were established as clinical and radiographic variables indicating periodontal regeneration and bone augmentation.

### 2.5. Quality Assessment

The bias risk in the human publications was evaluated by two reviewers (S.D. and P.K.) with the use of the Newcastle–Ottawa Scale tool for cohort non-randomized trials [36]. This tool permits quality assessment of three different domains (selection, comparability, and outcome) followed by some questions to be answered in each one. An answer to each question can provide one or two stars, and it can be characterized as good, fair, or poor quality depending on the number of stars provided. Regarding the included animal studies, two reviewers assessed the quality using SYRCLE’s risk of bias tool for animal studies. This scale includes 10 entries, and half of them are in accordance with the domains in the Cochrane Risk of Bias (RoB) tool [37]. 

## 3. Results

**Figure 3:**
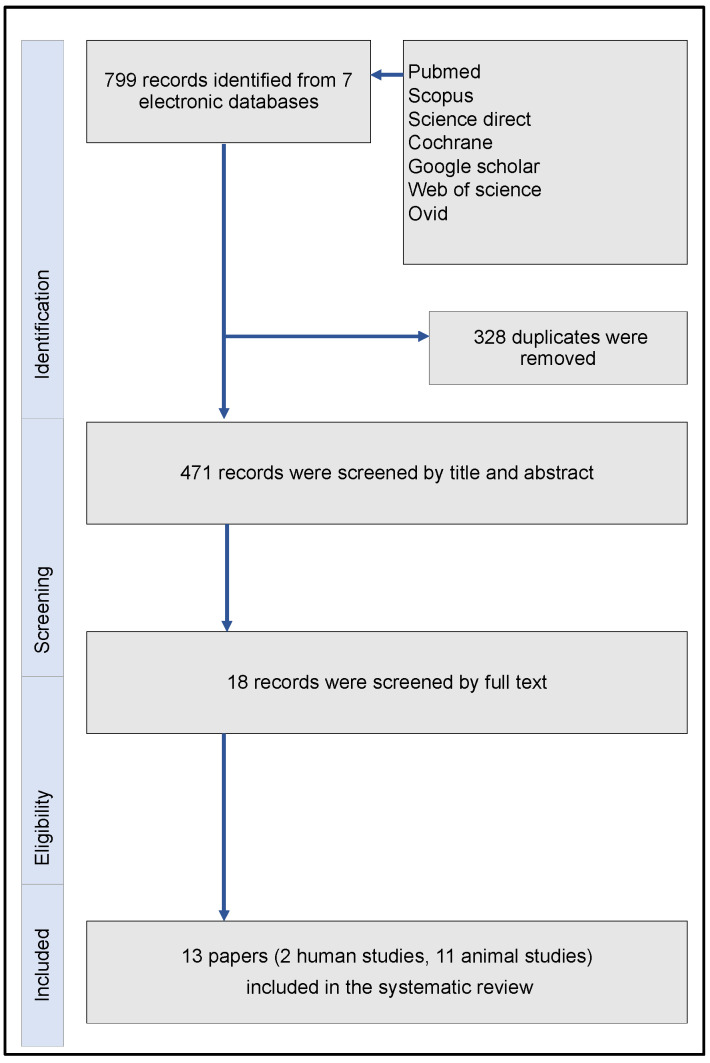
Flowchart of the systematic review, in accordance with the PRISMA guidelines.

Initially, the search strategy reported 799 articles. Following the removal of duplicates, these articles were decreased to 471 publications. Screening steps allowed for 18 trials be evaluated on a full-text level. Finally, merely two human [38,39] and 11 eleven animal studies [40,41,42,43,44,45,46,47,48,49,50] fulfilled all inclusion and exclusion criteria. The flowchart of the aforementioned process was in accordance with the PRISMA guidelines (Figure 3). Table 1 and Table 2 summarized all main characteristics of the included clinical human and animal studies, respectively.

In terms of human studies (Table 1), one was focused on periodontal regeneration in patients with periodontitis [39], while the other addressed bone augmentation before dental implant placement in periodontal patients with partial edentulism [38]. In the investigation conducted by Baba et al. [39], employing 3D scaffolds combined with platelet-rich plasma, a noteworthy enhancement in clinical attachment level, reduction in pocket depth, and bone growth were observed, resulting in an average linear bone growth (LBG) of 4.7 mm during the 36-month follow-up. Additionally, Mangano et al. [38] demonstrated in their study that the utilization of 3D scaffolds resulted in improved osteogenetic combability of bone defects in 8 months. Specifically, the histomorphometric analysis of bone cores revealed 34.9% (±4.2) new bone, 26.3% (±2.8) biomaterial, and 38.8% (±4.7) marrow spaces.

The follow-up period was significantly shorter in animal studies (up to 3 months) than in human studies (Table 1 and Table 2). Among the animal studies [40,41,42,43,44,45,46,47,48,49,50], three were conducted on rabbits, six on rats, one on mice, and one on beagle dogs. Although outcome criteria varied widely among the animal studies, all reported a trend of improvement in bone regeneration, as shown in Table 2. 

Among all the included research papers, there was notable variation in the types of 3D scaffolds employed, and a variety of additional agents were used as adjunct biomolecules in some, as presented in Table 1 and Table 2.

A meta-analysis was precluded from being conducted for several reasons. No study among those included were categorized as having a low risk of bias, and there was considerable heterogeneity across all aspects of the study design. 

The quality assessment evaluated with the Newcastle–Ottawa Scale for non-randomized clinical trials for each human trial is presented in Table 3 [36]. At the level of overall quality, both studies were reported as being poor quality due to zero stars in the comparability domain. The quality assessment evaluated with the use of SYRCLE’s risk of bias tool for each animal study is presented in Table 4 [37]. Only one of the animal studies [50] provided information on the random outcome assessment (detection bias) and none on blinding (detection bias). Moreover, only four of the studies [43,47,48,50] were assessed as having a low risk of bias regarding random housing (performance bias), while the rest reported no information for this domain. Regarding SYRCLE’s risk of bias tool, it is not recommended to calculate a summary score for each individual study [37].

## 4. Discussion

The primary goal of periodontal therapy has consistently centered on restoring the complete architecture of the periodontal complex. This intricate approach encompasses the generation of new bone, the formation of new cement on treated dental roots, and the establishment of periodontal fibers connecting the root surface to the alveolar bone. Typically, predictable periodontal regeneration is indicated in three-wall defects and class II furcation defects. It is noteworthy that conventional periodontal techniques, whether surgical or non-surgical, have been observed to fall short in accurately reproducing the original architecture and role of periodontal tissue. This realization has spurred the evolution of surgical periodontal regenerative techniques, with the goal of achieving complete and reliable periodontal regeneration. Among these techniques, guided tissue regeneration (GTR) procedures have gained widespread acceptance and remain fundamental in periodontal regenerative medicine. Regenerative medicine, as a promising avenue for personalized periodontitis treatment, encompasses a variety of approaches such as bone grafts, substitutes, guided tissue regeneration membranes, and biological factors. Combinations of these methods have demonstrated nearly identical success rates. However, the literature emphasizes that the morphology of intrabony defects, particularly factors like defect depth, angle, and the number of walls evident on radiographic images, plays a pivotal role in facilitating predictable periodontal regeneration. Understanding the intricacies of defect morphology is crucial in making informed decisions regarding flap design and may influence the selection of biomaterials for use [23]. The advent of CAD/CAM technology introduces innovative solutions by first identifying intrabony defects and their morphology, followed by manufacturing precise scaffold structures tailored to match these defects. Three-dimensional scaffold printing constitutes an interesting alternative to traditional periodontal regeneration techniques. Employing computer-aided design and manufacturing following CT scanning, this approach generates a 3D model. After the creation of the model, customized scaffolds can be produced. These scaffolds in three dimensions aptly conform to the dimensions and contours of the patient’s bone defect, enhancing adhesion, proliferation, cell differentiation, and, consequently, tissue regeneration. The objective of this approach is to substitute the defect with viable and operational tissue that closely resembles the original tissue. These scaffolds may consist of one or a combination of materials, including natural polymers, synthetic polymers, or bioceramics. Moreover, scaffolds can be combined with stem cells and/or growth factors in order to enhance bioactivity and augment regenerative potential. This innovative technique appears to possess the capabilities needed to facilitate comprehensive periodontal regeneration [51].

To the authors’ knowledge, this systematic review is an original contribution assessing the effectiveness of 3D scaffolds in regenerating periodontal intrabony defects in both human and animal subjects. The study systematically reviewed two human studies [38,39] and eleven animal studies [40,41,42,43,44,45,46,47,48,49,50] that met the pre-established inclusion criteria. Regrettably, due to the substantial heterogeneity among experimental protocols, encompassing variations in animal species, study design, scaffold types and compositions, adjunct biomolecules, and notably, measurement methods and outcome criteria, a meta-analysis proved unfeasible. The diverse nature of these variables underscores the need for standardized methodologies in future studies to enable more comprehensive analyses and comparisons across different research efforts.

In summary, our findings indicate that 3D-printed scaffolds are user-friendly, provide a well-suited fit to the defect, foster effective healing, and generally lead to superior outcomes in relation to periodontal parameters. Over the past few years, tissue engineering has increasingly focused on bone reconstruction utilizing 3D scaffolds to achieve optimal biological and mechanical outcomes [32]. 

The core advantages of these bone-grafting materials should ideally include minimal immunogenicity, bioactivity, and seamless interaction with host tissues [28]. Notably, a 3D scaffold must offer provisional skeletal support until the development of new bone tissue, featuring low or negligible antigenicity, structural integrity, and porosity to facilitate efficient cell and nutrient dissemination across the entire framework. In addition, the 3D scaffold should be biodegradable—an indispensable factor for absorption by neighboring tissues, negating the necessity for surgical extraction. Concurrently, the rate of resorption should be gradual, ensuring degradation aligns with the concurrent formation of new tissue. In the studies considered, there was diversity observed in the types of bioprinted scaffolds.

Bioprinting poses a significant challenge in the careful selection of materials, given their inherent complexity and often conflicting nature. The essential components for bio-printing technology encompass a polymer solution, viable cells, and 3D printers, collectively forming bioink [28]. Several considerations influence the choice of these components. For instance, the selected polymer solution should exhibit attributes such as low viscosity, low stiffness, and low cross-linking in order to facilitate cell migration, nutrient and oxygen diffusion, and ultimately the formation of new tissue. Conversely, optimal mechanical properties, dimensional accuracy, and improved structural features necessitate polymers with higher viscosity, stiffness, and rapid gelation. Striking a balance between the structural, physico-mechanical, and biological properties of the chosen polymeric materials is crucial to ensure the bioprinting of constructs with heightened cell viability [28,52]. Different materials used for 3D printing can be categorized as natural materials (e.g., gelatin, collagen), synthetic materials (e.g., PLGA, PCL) and ceramics (e.g., HA, β-TCP) [52]. Within the reviewed literature, divergence was observed in the array of bioprinted scaffold types. Specifically, in the studies under scrutiny, one human investigation [38] and one animal inquiry [45] opted for porous blocks composed of hydroxyapatite (HA). Hyaluronic acid (HA), heralded for its natural presence in the extracellular matrix (ECM) and distinctive characteristics, encompassing elevated viscoelasticity, biocompatibility, and degradability, has emerged as a promising material in the realm of bioprinting. Notably, HA constitutes 11% of the overall polymer distribution in the preparation of bioink. It is noteworthy that HA constitutes 11% of the overall polymer distribution during bioink preparation. Nevertheless, it is crucial to recognize that the heightened hydrophilicity inherent to HA might compromise the stability of the bioprinted constructs, thereby constraining its potential applications [28]. Furthermore, a pair of animal studies [42,43] integrated scaffolds fashioned from polycaprolactone (PCL). PCL, a biodegradable synthetic polyester extensively utilized in the bioprinting sphere, showcases improved stiffness and elasticity. It facilitates the growth of human chondrocytes, maintaining their cell morphology, viability, gene expression, and the ability for matrix production [28]. Chen et al. [44] endeavored to create a hybrid mesoporous bioceramic material comprising hydroxyapatite (HA), calcium sulfate (CS), and polycaprolactone (PCL) with the aim of optimizing the composite. The rationale behind incorporating CS in the combination was to address the limitations of HA, specifically stabilizing degradation rates and release rates [44]. They concluded that HA/CS scaffolds demonstrated both good degradation and mechanical strength [44]. In a singular animal investigation, scaffolds falling under the classification of natural polymers were integrated, particularly within the subset of gelatins [46]. Gelatin has proven to be highly effective in the formulation of bioink for bioprinting materials, owing to its distinctive characteristics, including elevated biocompatibility, biodegradability, significant cross-linking potential, and enhanced thermal stability within a physiological environment [28,46].

Baba et al. utilized a scaffold in their study, constructed from biodegradable poly-L-lactic acid resin fibers that were enhanced with stem cells [39]. This scaffold was produced through the electrospinning technique, where a high voltage supply generated an electric field, transforming the initial polymer into thin fibers. This process allowed for precise control over pore size, porosity, fiber thickness, and internal and external geometry [51]. The electrospinning manufacturing technique was also employed in another study involving PCL scaffolds [49]. In contrast, Park et al., in both of their studies, utilized fibers as a scaffold but manufactured them through stereolithography [40,41], marking the first application of this technique to 3D printing. Stereolithography utilizes a reservoir containing a photosensitive polymer, which undergoes curing through a light source in a process known as photopolymerization. A computer-controlled mirror guides a laser to intricately outline the design pattern within the photosensitive polymer. The areas where the laser interacts lead to the solidification of the liquid. Following the completion of each layer, the platform ascends in accordance with the designated layer thickness, while an additional liquid polymer flows beneath the recently printed layer. Qin et al. [48] also opted for ceramic scaffolds fabricated through stereolithography. In a different study, titanium scaffolds were created using the powder bed fusion technique [47]. In this procedure, the powder bed is situated within an inert atmosphere or partial vacuum to protect the molten metal. An energy source, either a laser or an electron beam, meticulously scans each layer of the evenly spread powder, selectively melting the material based on the part’s cross-section derived from the digital model. Subsequent to scanning a layer, the building chamber piston descends, while the powder chamber piston ascends in accordance with the defined layer thickness. A coating mechanism or roller deposits powder across the build chamber, which is then subjected to scanning by the energy source. This sequence is repeated layer by layer until the entire part is fabricated. The outcome is a powdered structure, and the final part remains concealed until excess powder is eliminated [52]. The same fabrication procedure was applied in another study for the manufacturing of ceramic scaffolds [50].

Since this is a relatively new approach, there are limited studies on humans, as revealed by the systematic review. Therefore, animal studies can provide insights into designing more effective and appropriate studies for humans. Animal models play a crucial role in evaluating the safety and functionality of innovative therapeutic approaches, offering an in-depth insight into their suitability for transitioning from laboratory research to clinical applications. While there is no perfect animal model for assessing the effectiveness of biomaterials and tissue engineering constructs in healing bone defects, it is highly desirable to have models that share similarities with human bone in terms of molecular, cellular, structural, and mechanical characteristics and have sizes compatible with the experimental design requirements [53,54]. In this systematic review, various animal models were identified and reported. Notably, the most frequently utilized ones included rats (six instances) and rabbits (three instances). Rats and rabbits are often favored due to their cost-effectiveness and ease of handling, manipulation, and maintenance [52,53]. However, it is important to acknowledge that bone micro- and macrostructure, as well as the bone-healing process, can vary significantly among different species. Additionally, within the same species, variations may occur depending on factors such as age, gender, overall systemic health, and biomechanical constraints [53,55]. Therefore, when transferring bone research findings from experimental animal models to the clinical setting, careful consideration is warranted due to the pronounced morpho-functional differences between small and large animals’ bone tissues [55].

Our systematic review also presents certain limitations. Given that the use of 3D printing in this scientific field represents a relatively new era, we anticipated a scarcity of studies specifically related to periodontal regeneration. Despite the absence of restrictions on publication year or language, only two human studies fulfilled the inclusion criteria, precluding the possibility of conducting a meta-analysis within this subgroup. Conversely, while a considerable number of animal studies were included, they involved different animals as experimental units. However, even among studies with the same experimental unit, significant methodological differences were evident, prohibiting the conduct of a meta-analysis.

## 5. Conclusions

This study demonstrates a consistent and significant enhancement in periodontal regeneration across all examined studies through the implementation of multidimensional 3D customized scaffolds. Despite the varied experimental protocols hindering meta-analysis, future investigations should draw upon the insights from our analysis to establish more standardized preclinical and clinical research protocols. This standardization would improve comparability and elevate the overall quality of foundational data, contributing to the strategic development of future clinical studies that comprehensively evaluate periodontal tissue regeneration techniques in terms of outcomes, reproducibility, and patient comfort. In general, periodontal tissue regeneration involves the formation of new bone, the development of new cement on treated dental roots, and the attachment of periodontal fibers between the root surface and alveolar bone. Guided tissue regeneration (GTR) stands out as the most evidence-based technique for promoting new attachments in periodontal therapy. GTR utilizes membranes as barriers to prevent epithelial and gingival connective tissue proliferation. Additionally, various growth factors have been employed to regenerate missing periodontal tissues. While these techniques are effective, they are often technique-sensitive and time-consuming. A drawback of using growth factors lies in their liquid or gel-like consistency, resulting in reduced space provision capacity. Therefore, scaffolds are frequently required as a vehicle. Given the increasing emphasis on personalized medicine and treatment, 3D personalized scaffolds can play a pivotal role in regenerative therapy by achieving both biological and mechanical tissue rehabilitation.

## Figures and Tables

**Table 1:** Main characteristics of the incorporated human studies.

Study [REF]	Study Design	Sample	Intervention	Type of Scaffold and Adjunct (If Any)	Follow Up	Outcome Criteria	Main Results
[38]	Prospective study	10 patients	3D HA scaffolds and graft for ridge augmentation and implant placement	Porous HA block	12 months	– Presence of pain, suppuration or exudation – Histological and histomorphometric evaluation	– Scaffolds were of satisfactory size, shape and appearance – Good match to the defect – Easy to handle – Reduced time of operation – Good healing
[39]	Prospective study	10 patients	(3D) woven-fabric composite scaffold and platelet-rich plasma for periodontal regeneration	3D woven-fabric of poly-L-lactic acidresin fibers combined to BMMSCs, PRP andhuman thrombin	36 months	– CAL – PD – LBG – Clinical mobility	– Improvement in CAL, PD, LBG – Average LBG of 4.7 mm after 36 months – Decreasing trend of clinical mobility

(HA): hydroxyapatite; (3D): three dimensional; (CAL): clinical attachment level; (PD); pocket depth; (LBG); linear bone growth; (β-TCP): β-tricalcium phosphate; (HA): hydroxyapatite; (PRP): platelet-rich plasma; (BMMSCs): bone marrow-derived mesenchymal stem cells.

**Table 2:** Main characteristics of the incorporated animal studies.

Study [REF]	Study Design	Sample	Intervention	Type of Scaffold and Adjunct (If Any)	Follow Up	Outcome Criteria	Main Results
[40]	Animal study	48 athymic rats	(1) hPDL-transplanted random-porous scaffolds; (2) hPDL-transplanted fiber-guiding scaffolds; (3) Ad-BMP-7-hPD seeded random-porous scaffolds; (4) hPDL (ligament interface) and Ad-BMP-7-hPDL (bone region) transplanted fiber-guiding scaffolds.	PCL Fiber-guiding scaffolds combined to BMP-7 and hPDL	6 weeks	Regeneration of ligament tissues	– Predictable oriented fiber architecture. – Greater control of tissue infiltration. – Better organization of ligament interface.
[41]	Animal study	48 athymic rats	(1) Amorphous PCL scaffolds without hPDL (2) Fiber-guiding scaffolds seeded with hPDL.	PCL Fiber guiding scaffolds combined to hPDL	6 weeks	Tissue healing and regeneration	– More mineralized tissues at 6 weeks. – Newly formed PDL fibers connected obliquely/perpendicularly to the root surface.
[42]	Animal study	24 mice	3D micropatterned polycaprolactone (PCL) scaffolds versus random-porous PCL.	PCL scaffolds	12 weeks	Collagen fiber thickness, cell alignment, nuclear elongation	At 6 weeks: 30 μm groove depth significantly enhanced oriented collagen fiber thickness, overall cell alignment, and nuclear elongation relative to 10 μm groove depth.
[43]	Animal study in rabbits	10 rabbits	(1) Negative control (control), (2) PCL block (PCL), (3) PCL mixed with 10 wt% β-TCP (PCL/β-TCP), and (4) PCL/β-TCP plus collagen membrane (PCL/β-TCP + M).	PCL scaffolds combined to β-TCP	8 weeks	Inflammation, allergic reaction, postoperative bleeding, infection, TAV (mm3), NBV (mm3)	Group 4 showed the highest TAV and NBV at 8 weeks, but there was no sign. difference among four groups. Histomorphometrically, Groups 2,3,4 showed significantly higher TAV compared to the control. NBV deep inside the scaffold increased hydrophilicity, and osteoconductivity was observed only in Group 3.
[44]	Animal study	Rabbits (exact number not reported)	HA; HA/VEGF; HACS; HACS/VEGF.	HACS scaffolds combined toVEGF	8 weeks	Osteoinduction and osteogenesis abilities	Excellent osteoinduction and osteogenesis.
[45]	Animal study	14 rats	In each animal, one defect with hydroxyapatite-based scaffold and one defect unfilled.	HA scaffolds	4 weeks	Osteoinduction and osteogenesis abilities	Most scaffolds fit the defects well. Type I collagen, VEGF, and Cbfa1 upregulated day 7. De novo osteogenesis and scaffold–tissue integration by day 28. Entire mineralized tissue and newly formed bone significantly promoted (micro-CT and histologic analyses).
[46]	Animal study	24 rats	(1) No treatment. (2) PLGA/gelatin composite scaffolds. (3) PLGA/gelatin composite-MSNs scaffolds. (4) PLGA/gelatin composite-Cu@ MSNs scaffolds.	Cu@ MSNs PLGA/gelatin scaffolds	12 weeks	Periodontal regeneration	Cu@ MSNs- PLGA/gelatin scaffolds reported: – Reduced CEJ-ABC distance, increased new bone volume; – Complete periodontal regeneration.
[47]	Animal study	10 athymic rats	G1: Titanium scaffold, collagen printing and cell seeding. G2: Titanium scaffold, collagen printing, FGF2 and cell seeding. G3: Titanium scaffold and cell printing. G4: Titanium scaffold, FGF2 and cell printing.	Titanium scaffolds combined to FGF2	6 weeks	Periodontal regeneration, new bone formation	Fibrous connective tissue not observed in G1 and G2 groups. Fibrous connective tissue apparent in G3 and G4 groups. New bone observed on the titanium implant surfaces in G1 and G2 groups.
[48]	Animal study	24 rabbits	6 mol% Mg-substituted CSi (magnesium-substitutedcalcium silicate (CSi-Mg6)) scaffolds with different pore dimensions.	CSi-Mg6 scaffolds	12 weeks	Angiogenesis and osteogenesis abilities	The 600 μm group exhibited an evidently higher ratio of the newly (BV/TV), trabecular number (Tb. N) values and new bone ingrowth rate at 4–12 weeks post-implantation.
[49]	Animal study	12 rats	(1) No treatment. (2) PCL scaffolds. (3) F/CaP-coated PCL scaffolds.	F/CaP-coated	6 weeks	Bone fill	F/CaP-coated scaffold group – Near complete bone coverage at 6 weeks. – Regeneration of new alveolar bone. cementum, and PDL at 3 weeks. – More organized periodontium at 6 weeks.
[50]	Animal study	Beagle dogs (exact number not reported)	(1) No treatment. (2) BCG scaffolds. (3) Mo-BCG scaffolds.	Mo-BCG scaffolds	8 weeks	Bone and ligament regeneration	– Larger amounts of new bone. – Functional PDL. – Newly formed cementum.

(CAL): clinical attachment level; (3D): three dimensional; (PD); pocket depth; (LBG); linear bone growth; (β-TCP): β-tricalcium phosphate; (hPDL): human periodontal ligament; (BMP): bone morphogenetic protein; (AdBMP-7): adenovirus-encoding BMP-7; (PCL): polycaprolactone; (M): collagen membrane; (TAV in mm^3^): total augmented volumes; (NBV in mm^3^): new bone volume; (HACS): hydroxyapatite/calcium sulfate; (VEGF): vascular endothelial growth factor; (CT): computed tomography; (Cbfa1): core-binding factor alpha-1; (Cu@MSNs): copper-loaded mesoporous silica nanoparticles; (PLGA/gelatin): poly(lactic-co-glycolic acid)/gelatin; (CSi-Mg6): magnesium-substituted calcium silicate; (Mo-BCG): molybdenum-containing bioactive glass ceramic; (F/CaP): fluorinated calcium phosphate; (BV/TV): bone volume to total volume; (Tb.N): trabecular number; (RV/TV): residual volume to total volume; (PDL): periodontal ligament; (BCG): bioactive glass ceramic; (FGF2): fibroblast growth factor 2; (CEJ): cementoenamel junction; (ABC): alveolar bone crest. (HA): hydroxyapatite; (PRP): platelet-rich plasma.

**Table 3:** Assessment of bias risk in the enlisted human studies using the Newcastle–Ottawa Scale for non-randomized clinical trials.

Author (Year)	Selection	Comparability	Outcome	Overall Evaluation
[38]	٭٭٭	-	٭٭٭	poor
[39]	٭٭٭	-	٭٭٭	poor

Good quality: 3 or 4 stars in the selection domain AND 1 or 2 stars in the comparability domain AND 2 or 3 stars in the outcome/exposure domain; fair quality: 2 stars in the selection domain AND 1 or 2 stars in the comparability domain AND 2 or 3 stars in the outcome/exposure domain; poor quality: 0 or 1 star in the selection domain OR 0 stars in the comparability domain OR 0 or 1 star in the outcome/exposure domain.

**Table 4:** Assessment of bias risk in the enlisted animal studies using SYRCLE‘s risk of bias tool.

Author (Year)	Sequence Generation (Selection Bias)	Baseline Characteristics (Selection Bias)	Allocation Concealment (Selection Bias)	Random Housing (Performance Bias)	Blinding (Performance Bias)	Random Outcome Assessment (Detection Bias)	Blinding (Detection Bias)	Incomplete Outcome Data (Attrition Bias)	Selective Outcome Reporting (Reporting Bias)	Other Sources of Bias (Other)
[40]	Low risk	Low risk	Low risk	Unclear	Low risk	Unclear	Unclear	Low risk	Low risk	High risk
[41]	Low risk	Low risk	Low risk	Unclear	Low risk	Unclear	Unclear	Low risk	Low risk	High risk
[42]	Low risk	Low risk	Low risk	Unclear	Low risk	Unclear	Unclear	Low risk	Low risk	Low risk
[43]	Low risk	Low risk	Low risk	Low risk	Low risk	Unclear	Unclear	Low risk	Low risk	Low risk
[44]	Low risk	Low risk	Low risk	Unclear	Low risk	Unclear	Unclear	Low risk	Low risk	High risk
[45]	Low risk	Low risk	Low risk	Unclear	Low risk	Unclear	Unclear	Low risk	Low risk	Low risk
[46]	Low risk	Low risk	Low risk	Unclear	Low risk	Unclear	Unclear	Low risk	Low risk	High risk
[47]	Low risk	Low risk	Low risk	Low risk	Low risk	Unclear	Unclear	Low risk	Low risk	High risk
[48]	Low risk	Low risk	Low risk	Low risk	Low risk	Unclear	Unclear	Low risk	Low risk	Low risk
[49]	Low risk	Low risk	Low risk	Unclear	Low risk	Unclear	Unclear	Low risk	Low risk	High risk
[50]	Low risk	Low risk	Low risk	Low risk	Low risk	Low risk	Unclear	Low risk	Low risk	High risk

## Data Availability

Data are contained within the article.
